# The Nexus of Practice Environment, Management, and Nursing Competency: A Cross‐Sectional Study of Community Nursing Services in Saudi Arabia’s Northern Border Region

**DOI:** 10.1155/jonm/1916013

**Published:** 2026-10-04

**Authors:** Fadiyah Jadid Alanazi, Amal Ahmed Elbilgahy, Mohamed Yehia Ali Mohamed, Bindu Bharathi, Fathia Ahmed Mersal

**Affiliations:** ^1^ Public Health Nursing Department Faculty of Nursing Northern Border University Arar Saudi Arabia nbu.edu.sa; ^2^ Maternal & Child Health Nursing Department Faculty of Nursing Northern Border University Arar Saudi Arabia nbu.edu.sa; ^3^ Pediatric Nursing Department Faculty of Nursing Mansoura University Mansoura Egypt mans.edu.eg; ^4^ Community Health Nursing Department Faculty of Nursing Cairo University Cairo Egypt cu.edu.eg

**Keywords:** community health nursing, cross-sectional study, mediation analysis, nursing competency, nursing management, nursing practice environment, primary health care, Saudi Arabia

## Abstract

**Aim:**

This study aimed to examine the associational role of the nursing practice environment (NPE) in the relationship between nursing management practices (NMP) and community health nursing competency (CCNC) among primary healthcare (PHC) nurses in Saudi Arabia.

**Background:**

Although the NPE is established as a determinant of care quality and patient safety in hospital settings, its function as an organizational mechanism linking managerial practice to nursing competency in community and PHC contexts remains underinvestigated, particularly within administratively peripheral regions undergoing health‐system transformation.

**Methods:**

A descriptive, cross‐sectional, correlational survey was conducted with 205 registered nurses working in PHC centers across the Northern Border Region (Arar), Saudi Arabia. Data were collected using a structured questionnaire incorporating an adapted quality of nursing work life subscale set, the practice environment scale of the nursing work index (PES‐NWI), and the community care nursing competence (CCNC) scale. Reporting followed the STROBE guidance. Normality was assessed via the Shapiro–Wilk test; parametric analyses were supplemented with nonparametric checks. Pearson correlations, hierarchical multiple regression, and bias‐corrected bootstrap mediation analysis (5000 resamples) were performed. Common method bias was assessed using Harman’s single‐factor test and a marker‐variable procedure; variance inflation factors (VIFs) were computed for all regression models.

**Results:**

All three constructs were strongly and positively associated (*r* = 0.76–0.79, *p* < 0.001). The NMP and NPE composites jointly explained 71.5% of the variance in CCNC (*F* [10, 191] = 44.62, *p* < 0.001), with NMP (beta = 0.47) and NPE (beta = 0.38) as substantial independent predictors. VIF values did not exceed 4.2, indicating acceptable multicollinearity. Harman’s single‐factor test extracted a first factor accounting for 43.6% of the total variance, below the 50% threshold, and the marker‐variable test yielded a change in fit of delta *R* ^2^ = 0.002, suggesting that common method variance did not critically inflate observed associations. The NPE significantly and partially mediated the NMP‐CCNC pathway (indirect effect = 0.33, 95% CI [0.11, 0.52]; Sobel *Z* = 6.15, *p* < 0.001), accounting for approximately 38.4% of the total effect. Consistent with the cross‐sectional design, these findings reflect association rather than causal pathways.

**Conclusions:**

CCNC is associated with both NMP and the practice environment, with the NPE serving as a significant statistical intermediary in this pathway. These associations are consistent with organizational frameworks positing that work context conditions translate managerial inputs into professional capability.

**Implications for Nursing Management:**

Strengthening unit‐level management capacity and the practice environment represents a potentially feasible and evidence‐informed strategy for advancing community nursing competency in PHC settings, particularly in geographically peripheral regions undergoing reform. Longitudinal and experimental designs are needed to establish directionality.

## 1. Introduction

Community nursing services represent a cornerstone of effective and equitable healthcare delivery worldwide, serving as the first point of contact for individuals, families, and communities within primary healthcare (PHC) systems. As healthcare systems globally shift their emphasis from hospital‐centric models to community‐based care, the role of community health nurses has expanded beyond traditional boundaries to encompass health promotion, disease prevention, chronic disease management, and the coordination of complex care across settings. This paradigm shift recognizes that a substantial proportion of healthcare needs can be efficiently and effectively addressed at the community level, reducing unnecessary hospital admissions, improving patient outcomes, and optimizing resource utilization [1, 2]. In this context, understanding the organizational factors that enable or constrain community nursing services, particularly the interplay between the practice environment and management practices and their associations with nursing competency, has emerged as a critical priority for health system strengthening and policy development.

The nursing practice environment (NPE) has been extensively studied in hospital settings and is recognized as a construct that fundamentally covaries with nursing‐care outcomes. A favorable NPE is characterized by adequate staffing and resources, strong nurse–physician collaboration, nurse participation in institutional decision‐making, and supportive managerial leadership. Although NPE has traditionally been linked to quality of care and patient outcome indicators, growing evidence suggests that it may also support nurses’ professional performance and competency. However, this relationship remains underexplored in Indian community and PHC settings. However, most of this evidence is derived from acute care hospital contexts, leaving a notable gap in our understanding of how NPE operates within PHC and community nursing settings, where the nature of work, patient populations, and organizational structures differ substantially from those in inpatient environments [3].

Nursing management practices (NMP) constitute a second critical determinant of nursing service quality, encompassing leadership approaches, resource allocation, staff development, and the implementation of quality improvement initiatives. Effective nursing management creates organizational conditions that enable frontline nurses to deliver safe, effective, and patient‐centered care [4]. A systematic review examining the impact of management strategies on nursing service quality found that transformational leadership, professional governance, and total quality management were consistently associated with improvements in patient satisfaction, staff commitment, and organizational efficiency [5]. Similarly, research examining nurse managers’ leadership has documented positive associations between effective unit‐level management and better patient outcomes, including fewer adverse events, increased error reporting, and higher care quality ratings [6]. These findings underscore the pivotal role of nursing management as an enabling factor associated with the daily realities of clinical practice and the quality of care patients receive.

The nexus between the practice environment and management is particularly salient because managerial decisions are closely associated with the conditions under which nurses work. Nurse managers occupy a unique position within healthcare organizations, serving as a bridge between strategic organizational priorities and frontline clinical operations. Their leadership style, resource allocation decisions, communication practices, and support for professional development are associated with the practice environment that staff experience daily. Conversely, the practice environment, characterized by factors such as staffing adequacy, autonomy, and collegial relationships, is associated with the relationship between management practices and care quality outcomes [7–9]. Understanding this dynamic interplay is essential for designing effective interventions to improve community nursing services, as efforts focused exclusively on management without addressing environmental constraints, or vice versa, are unlikely to achieve sustained improvements.

Theoretically, Donabedian’s structure‐process‐outcome (SPO) model provides an apt organizational lens for this inquiry [10]. Within the SPO framework, NMP can be conceptualized as structural inputs, encompassing staffing policies, governance mechanisms, and leadership systems, while the practice environment represents the processual conditions through which these structures are operationalized. Community health nursing competency (CCNC), in turn, constitutes the professional outcome. This triadic framework posits that structural inputs are associated with outcomes both directly and through the mediating process of the work environment, an architecture directly mirrored in the mediation model tested here. Importantly, a cross‐sectional design can identify associations consistent with this framework but cannot establish temporal or causal ordering among the constructs.

Saudi Arabia is currently undergoing a profound transformation of its healthcare system as part of the ambitious Vision 2030 national reform agenda. The Ministry of Health has launched multiple initiatives to strengthen PHC as the foundation of the health system, including the Nartqy program to encourage quality improvement projects within healthcare centers and the Adaa monitoring dashboard to track service performance and facilitate data‐driven decision‐making [2]. Digital transformation efforts have introduced services such as Mawid for electronic appointment booking and Wasfati for electronic prescription management, alongside the implementation of a nationwide electronic medical record system [2]. These reforms recognize that a strong PHC system, anchored by capable community nursing services, is essential for achieving universal health coverage, improving population health outcomes, and containing healthcare costs. However, the pace and scale of these changes also present significant implementation challenges that require careful study.

Despite these ambitious reforms, the nursing profession in Saudi Arabia faces persistent challenges that threaten the quality and sustainability of community nursing. The country maintains a high ratio of expatriate nurses, creating a complex and multicultural workforce with diverse educational backgrounds, clinical experience, and cultural competencies [11]. A recent systematic review documented consistent positive associations between NPE and job satisfaction among nurses in Saudi Arabian hospitals, but it also identified methodological limitations and highlighted the need for more rigorous longitudinal studies, particularly in primary care settings. Staffing and resource adequacy (SRA) remain problematic in many facilities, with studies documenting poor perceptions of resource availability among nurses, which, in turn, are associated with lower job satisfaction, performance, and care quality delivered to patients [12, 13]. The challenges of limited nursing school capacity, labor market fragmentation, a shortage of nurses in rural areas, and gender‐related obstacles further compound these difficulties [14].

The Northern Border Region of Saudi Arabia presents a distinctive and understudied context for examining community nursing services. Located in the northernmost administrative zone of the kingdom, bordering Jordan to the north‐west and Iraq to the north‐east, the region is one of Saudi Arabia’s least densely populated and most geographically isolated provinces. Its capital, Arar, functions as the primary administrative and healthcare hub for a dispersed population spread across vast desert terrain. Compared with metropolitan clusters such as Riyadh or Jeddah, the Northern Border Region has historically received fewer healthcare investment resources proportionally, faces acute challenges in recruiting and retaining skilled health professionals, including nurses from outside the region, and relies disproportionately on PHC centers as the principal or sole point of contact with the health system for most residents. Regional nurse‐to‐population ratios fall below national averages, and logistical barriers limit referral networks to secondary and tertiary facilities [15]. These structural disparities mean that the quality of unit‐level management and the immediate practice environment may carry disproportionate weight in determining whether community health nurses can practice competently, since the compensatory mechanisms available in better‐resourced urban settings (specialist supervision, rapid referral, and dense professional networks) are less accessible here.

Recent studies conducted in the Northern Border Region have examined specific aspects of nursing practice, including cultural competence among pediatric nurses and organizational commitment among nursing staff; however, no comprehensive investigation has addressed the interconnected domains of practice environment, management practices, and nursing competency within community nursing services in this region [2, 16]. This represents a critical evidence gap, as the challenges facing community nursing in peripheral and border regions likely differ substantially from those in urban centers, necessitating context‐specific research to inform tailored policy responses.

The NPE is most measured using the practice environment scale of the nursing work index (PES‐NWI), a validated 31‐item instrument derived from the organizational characteristics of Magnet hospitals [17]. The PES‐NWI encompasses five dimensions: staffing and resource adequacy (SRA); nursing foundations for quality of care (NFQC); nurse manager ability, leadership, and support (NMALS); nurse participation in hospital affairs (NPHA); and collegial nurse–physician relations (CNPR). In the Saudi PHC context, evidence has highlighted significant deficiencies in SRA, with overall NPE scores reflecting unfavorable conditions [12]. These findings underscore the need to investigate further how NMP are associated with specific dimensions of the practice environment within community and PHC settings.

## 2. Research Gap and Study Purpose

The present study addresses the identified gaps in knowledge by examining the associational nexus between NMP, the NPE, and CCNC in Saudi Arabia’s Northern Border Region. Employing a cross‐sectional design, the study integrates the assessment of the practice environment using the PES‐NWI and explores management practices and nursing competency among registered nurses (RNs), aiming to generate actionable evidence to inform policy and practice. By focusing specifically on the Northern Border Region, the research produces contextually relevant findings that can guide targeted interventions to strengthen community nursing services in this geographically peripheral and under‐researched area, while also contributing to the broader international literature on the determinants of nursing competency in PHC settings. It is important to note at the outset that the cross‐sectional design allows the examination of statistical associations and an associational mediation model consistent with the theoretical framework; it does not permit causal inferences about the direction or mechanism of observed relationships.

## 3. Study Objectives and Hypotheses

### 3.1. Study Objectives

Grounded in organizational and nursing management theory and anchored in the Donabedian’s SPO framework, this study aimed to examine the structural associations among NMP, the NPE, and CCNC. Specifically, this study sought to

1. Assess the levels of NMP, NPE, and CCNC among PHC nurses in Arar.
2. Examine the bivariate associations between NMP, NPE, and CCNC.
3. Evaluate the predictive associations of NMP and NPE with CCNC using multivariate regression models.
4. The mediating role of NPE in the pathway between NMP and CCNC was tested using a theoretically specified mediation model with bootstrap inference.

### 3.2. Hypotheses

Based on the proposed conceptual framework and prior empirical evidence, the following hypotheses were formulated. Consistent with the cross‐sectional design, the hypotheses are framed as propositions about statistical associations rather than causal pathways.

 H1: NMP are positively associated with the NPE.
 H2: NPE statistically mediates the association between NMP and CCNC.

## 4. Methodology

### 4.1. Research Design

A descriptive, cross‐sectional, correlational survey design was employed to examine the associational role of the NPE in the relationship between NMP and CCNC among RNs in the Northern Border Region of Saudi Arabia. A cross‐sectional design was deemed appropriate given the study’s aim to quantify the strength and direction of associations among study constructs at a single point in time and to test a theoretically driven mediation model without manipulation of variables [18]. Reporting adheres to the Strengthening the Reporting of Observational Studies in Epidemiology (STROBE) statement for cross‐sectional studies to enhance methodological transparency and reproducibility [19]. Consistent with the observational design, all findings are interpreted as associational; cross‐sectional data do not permit conclusions about causal ordering, temporal sequence, or directionality among constructs.

### 4.2. Setting

The study was conducted across PHC Centers and affiliated community‐based clinical facilities providing community nursing services in Arar city, the administrative capital of the Northern Border Region of Saudi Arabia. As detailed in the Introduction, the Northern Border Region is a geographically peripheral zone characterized by sparse population density, dependence on PHC as the principal healthcare entry point, a multinational nursing workforce, and a healthcare infrastructure that lags behind metropolitan regions in staffing and resource endowment. This regional context is directly relevant to the study: the findings are not intended to generalize better‐resourced metropolitan or hospital settings but rather to provide an evidence base for improving community nursing in similar peripheral PHC environments.

### 4.3. Participants

#### 4.3.1. Target Population and Sampling

The target population comprised RNs actively employed in community nursing and PHC roles in Arar. A nonprobability convenience sampling technique was employed, which is a pragmatic and widely accepted approach in nursing workforce research, where probability sampling frames are often unavailable [20]. The convenience sampling approach limits the external generalizability of the findings beyond the study region and PHC context, a limitation explicitly acknowledged in the discussion.

#### 4.3.2. Eligibility Criteria

The inclusion criteria were as follows: (a) RNs of any nationality currently employed in community/PHC settings in Arar; (b) a minimum of 6 months of clinical experience in the current role to ensure adequate familiarity with the practice environment; and (c) the ability to read either Arabic or English. The exclusion criteria were as follows: (a) nurses occupying purely administrative or nonclinical positions and (b) nurses with less than 6 months of community nursing experience.

#### 4.3.3. Sample Size Determination

Sample size was calculated a priori using G∗Power 3.1 [21] for a multiple linear regression model, assuming a medium effect size (*f* ^2^ = 0.15), an alpha level of 0.05, statistical power of 0.80, and up to eight predictors. This computation indicated a minimum requirement of approximately 100 participants. To ensure adequate power for the hierarchical OLS regression models with up to ten predictors reported herein, a supplementary calculation was performed assuming the same parameters (*f* ^2^ = 0.15, alpha = 0.05, power = 0.80, ten predictors), yielding a minimum of approximately 147 participants; the final analytic sample of *N* = 205 exceeds this threshold, providing adequate power with a margin. To accommodate potential nonresponse and to ensure sufficient statistical power for the planned mediation analysis with bias‐corrected bootstrapping, the recruitment ceiling was expanded. Invitations were disseminated to 274 eligible community/PHC nurses across participating facilities in Arar. Of these, 215 nurses returned the questionnaire, yielding a response rate of 78.5%. Following data screening, ten responses were excluded due to substantial incompleteness (> 20% missing items) or failure to meet eligibility criteria. The final analytic sample, therefore, comprised *N* = 205 community/PHC nurses, with an overall item‐level missingness of less than 0.01% (1 of 18,040 cells), well below conventional thresholds warranting imputation [22].

### 4.4. Instruments

A structured, self‐administered questionnaire comprising four sections was used to operate the study constructs. All non‐Arabic instruments were forward‐translated into Arabic by a bilingual nursing academic, back‐translated independently into English by a second bilingual expert who was blinded to the original text, and then reviewed by an expert panel of three bilingual nursing scholars to resolve discrepancies and ensure semantic, idiomatic, and conceptual equivalence, following Brislin’s [23] established protocol. The final Arabic versions were pilot tested for face validity, clarity, and cultural appropriateness before full deployment.

#### 4.4.1. Section 1: Sociodemographic and Professional Characteristics

This investigator‐developed section captured age, sex, nationality, highest academic qualification, total nursing experience (years), experience in the current community/PHC role (years), and facility type.

#### 4.4.2. Section 2: NMP

NMP were assessed using a 23‐item composite adapted from the work design and work context dimensions of the Quality of Nursing Work Life (QNWL) Survey [24]. It is important to clarify that this instrument was originally developed to measure nurses’ quality of work life rather than NMP per se. In the present study, the work design, work environment, work world (WW), and work–life balance dimensions were selected and adapted because they collectively capture the aspects of nurses’ immediate work context that are shaped by and reflective of the prevailing management practices at their unit, including the extent to which management provides autonomy, feedback, workload equity, resources, and a positive relational culture. While this operationalization represents a pragmatic and theoretically coherent adaptation, readers should note that it measures nurses’ perceptions of their work context as a proxy for management quality rather than directly assessing formal managerial behaviors or leadership style. Future studies should consider complementing this approach with validated nursing management‐specific instruments. Items were rated on a 6‐point Likert scale ranging from 1 (*strongly disagree*) to 6 (*strongly agree*), with higher scores reflecting more supportive managerial practice contexts. In the present sample, the composite demonstrated excellent internal consistency (Cronbach’s alpha = 0.939). Subscale reliabilities were acceptable to excellent: work design (alpha = 0.884), work environment (alpha = 0.920), and WW (alpha = 0.902); the work–life balance subscale yielded alpha = 0.730. The exceptionally high alpha values (several subscales exceeding 0.90) reflect the substantial thematic coherence of the adapted item sets and the homogeneous sample context; they also indicate that items within each dimension are measuring a common construct. As discussed further in the Limitations section, very high alpha may also signal some degree of item redundancy or narrow construct bandwidth. Nonetheless, the subscale‐level alpha remains within or just above the range conventionally considered optimal (0.70–0.95), and the composite is used as a summary measure of perceived work context quality rather than as a multidimensional scale.

#### 4.4.3. Section 3: NPE

The NPE was assessed using the PES‐NWI [17], one of the most widely validated and internationally recommended instruments for measuring nurse work environments, endorsed by the National Quality Forum. The original PES‐NWI was developed in hospital settings; the community/PHC adaptation employed in this study retained all 29 items that were contextually applicable to PHC settings, following a minor contextual refinement procedure (e.g., “hospital” was replaced with “health center” where appropriate). This adaptation was reviewed by the expert bilingual panel described above. The instrument captures five empirically derived subscales: (a) NPHA; (b) NFQC; (c) NMALS of nurses; (d) SRA; and (e) CNPR. Items were scored on a 4‐point Likert scale ranging from 1 (*strongly disagree*) to 4 (*strongly agree*). The total scale demonstrated outstanding internal consistency (alpha = 0.977), with strong subscale reliabilities (NPHA alpha = 0.951; NFQC alpha = 0.941; NMALS alpha = 0.933; SRA alpha = 0.929; CNPR alpha = 0.935). As noted above, alpha values exceeding 0.95 at the composite level may indicate some item redundancy. The composite total score was used as the primary NPE measure in analyses; subscale scores were retained for the purposes of descriptive reporting and to illuminate the correlational structure.

#### 4.4.4. Section 4: CCNC

Nursing competency was measured using the 31‐item CCNC scale developed by Kuo et al. [25], which comprises three theoretically and empirically distinct subscales: community care practice (15 items), communication (8 items), and management (8 items). Responses were captured on a 5‐point Likert scale ranging from 1 (not competent) to 5 (highly competent). Reliability in the current sample was excellent for the total scale (alpha = 0.978) and for all subscales (practice alpha = 0.959; communication alpha = 0.945; management alpha = 0.949). CCNC is a self‐rated competency instrument; therefore, scores reflect nurses’ self‐assessed rather than objectively observed competency. This distinction is acknowledged in the Limitations section.

### 4.5. Procedure

#### 4.5.1. Pilot Testing

Before full deployment, the questionnaire was pilot tested with 10 community nurses meeting the eligibility criteria to evaluate face validity, clarity, cultural appropriateness, and completion time (mean = 14 min). Pilot data were not included in the final analysis, and minor wording refinements were incorporated based on participant feedback.

#### 4.5.2. Data Collection

Data collection was performed within 2 months between February 20, 2026, and April 20, 2026, in PHCs and community healthcare facilities located in Arar, Northern Border Region, Saudi Arabia. With institutional approvals received, a mixed method was used to collect data to encourage participation and minimize the selection bias caused by using only one method. Eligible nurses were selected by examining PHC records and were asked to participate by institutional correspondence. The participation kit contained a participant information sheet containing detailed information about the purpose of the study, eligibility criteria, voluntary participation, confidentiality, and guidance on the completion of the questionnaire. There were two methods of participation offered. First, there was an opportunity to participate in the study by completing an online encrypted questionnaire that could be filled through a website link sent by the PHC administrator. Second, there was also a paper‐based questionnaire that could be filled in by those participants who wished to fill it in manually or did not have access to the online questionnaire.

Reminders were sent out at predetermined dates to enhance participation. Two weeks and 4 weeks after sending out the first letter with the invitation, nurses received reminders via institutional correspondence. Participants who completed the paper‐based questionnaire returned it through a sealed envelope at a predetermined collection point in their facility, while the online questionnaire was automatically recorded in the survey database. Personal identifiers were not requested in any of the questionnaires to guarantee participant anonymity. After the end of the data collection period, all returned questionnaires were checked for eligibility and missing data. In total, 274 eligible community and PHC nurses were invited to participate; 215 questionnaires were returned, which constituted a 78.5% response rate. After the data screening, 10 questionnaires were removed because of a substantial number of missing data (more than 20%) and ineligible criteria. Thus, 205 questionnaires were analyzed in total.

#### 4.5.3. Ethical Considerations

The study adhered to the principles outlined in the “Declaration of Helsinki [26].” Ethical agreement was granted from Northern Border University with the following number HAP‐09‐A‐043 with decision number 12/26/H, 14‐02‐2026. Ethical approval was obtained from the relevant Institutional Review Board (IRB) and the Directorate of Health Affairs of the Northern Border Region before data collection. A written information sheet describing the study purpose, voluntary nature, anonymity safeguards, data confidentiality, and participants’ right to withdraw without consequence preceded the questionnaire. Submission of the complete questionnaire constituted implied informed consent. No personally identifying information was collected, and electronic data were stored on password‐protected servers accessible only to the research team.

#### 4.5.4. Data Analysis

Data were analyzed using Python 3.11 (pandas, SciPy, and statsmodels), with statistical significance set at *p* < 0.05 (two‐tailed). Prior to inferential analyses, the data were screened for accuracy, completeness, and distributional assumptions using SPSS. Normality was assessed using skewness, kurtosis, the Shapiro–Wilk test, and visual inspection of histograms and Q‐Q plots. Although some variables demonstrated statistically significant departures from normality, the distributions exhibited only mild skewness and were considered acceptable for parametric analyses. Nonparametric tests were conducted as sensitivity analyses where appropriate. The internal consistency reliability of all scales and subscales was evaluated using Cronbach’s alpha coefficients, with values  ≥ 0.70 considered acceptable [27]. Continuous variables were summarized using means and standard deviations and medians and interquartile ranges, where appropriate. Categorical variables are reported as frequencies and percentages. Differences in study variables across sociodemographic characteristics were examined using Welch’s *t*‐tests and one‐way analysis of variance (ANOVA), with the Mann–Whitney *U* and Kruskal–Wallis *H* tests used to assess the robustness of the results. Pearson’s product‐moment correlation coefficients were calculated to examine the associations among the principal study variables.

Multiple linear regression analyses were performed to identify factors associated with CCNC, with nurse manager performance and NPE entered as the primary predictors and age, sex, educational level, and years of experience included as covariates. Multicollinearity was assessed using variance inflation factors and condition indices, and heteroscedasticity‐consistent (HC3) standard errors were applied where appropriate. The hypothesized mediating role of the NPE in the relationship between nurse manager performance and CCNC was examined using the PROCESS framework [28, 29] with 5000 bias‐corrected bootstrap resamples. Indirect effects were considered statistically significant when the 95% bootstrap confidence interval did not include zero, and the Sobel test was reported as a supplementary analysis [30]. Given the cross‐sectional design, the mediation findings were interpreted as evidence of an associational pathway rather than causality. Because all study variables were collected through self‐report measures at a single time point, potential common method bias was evaluated using Harman’s single‐factor test and a marker‐variable approach. The results of these analyses are reported in the Results section.

## 5. Results

Table 1 illustrates reliability coefficients and univariate descriptive statistics for composite scales and subscales. A total of *N* = 205 community/PHC nurses practicing in the Northern Border Region (Arar) of Saudi Arabia provided complete or near‐complete data. The sample was predominantly female (*n* = 146; 71.2%), with a mean age of *M* = 34.55 years (SD = 5.10; range = 25–51). Educationally, 50.2% (*n* = 103) held a diploma in nursing, 45.4% (*n* = 93) a bachelor of science in nursing (BSN), and 4.4% (*n* = 9) a master of science in nursing (MSN). The mean total nursing experience was 7.58 years (SD = 3.52), and the mean experience in the current community/PHC role was 5.42 years (SD = 3.22). Item‐level missingness was negligible (1 of 17,015 item responses; 0.006%), and person‐mean within‐subscale imputation was applied for this single missing value. Internal consistency was excellent for all three composites and uniformly strong across subscales. Shapiro–Wilk tests were statistically significant for all distributions (consistent with sensitivity to *N* = 205), but visual inspection confirmed approximate symmetry and mild negative skew, supporting the use of parametric procedures supplemented by nonparametric robustness checks. Harman’s single‐factor test extracted the first unrotated factor accounting for 43.6% of total variance across all scale items, below the conventional 50% threshold. The marker‐variable analysis yielded a change in *R* ^2^ of delta *R* ^2^ = 0.002 when the social desirability marker was added to the regression model, and the marker’s beta coefficient was not significant (beta = 0.04, *p* = 0.38).

**TABLE 1:** Reliability coefficients and univariate descriptive statistics for composite scales and subscales (*N* = 205).

Scale/subscale	*k* item	Alpha	*M*	SD	Median	Skew	Kurtosis	S‐W W (*p*)
Nursing management practices (NMP)	23	0.948	3.67	0.84	3.70	−0.65	2.39	0.939 (< 0.001)
Work–life balance (WLB)	5	0.818	3.54	0.92	3.60	−0.47	0.91	0.965 (< 0.001)
Work design (WD)	6	0.898	3.69	0.98	3.67	−0.39	0.97	0.965 (< 0.001)
Work environment (WE)	7	0.910	3.68	0.99	3.71	−0.36	0.84	0.969 (< 0.001)
Work world (WW)	5	0.902	3.79	1.06	3.80	−0.34	0.54	0.970 (< 0.001)
Practice environment scale (PES‐NWI)	29	0.977	3.67	0.91	3.62	−0.38	1.97	0.936 (< 0.001)
Nurse participation in affairs	9	0.951	3.63	0.99	3.56	−0.39	0.97	0.956 (< 0.001)
Foundations for quality of care	6	0.941	3.73	1.01	3.67	−0.41	0.94	0.960 (< 0.001)
Nurse manager ability/leadership	5	0.933	3.68	1.03	3.60	−0.25	0.83	0.965 (< 0.001)
Staffing and resource adequacy	4	0.929	3.60	1.09	3.50	−0.15	0.33	0.973 (< 0.001)
Collegial nurse–physician relations	5	0.935	3.71	1.06	3.60	−0.31	0.64	0.965 (< 0.001)
Community health nursing competency (CCNC)	31	0.978	3.75	0.91	3.68	−0.10	1.95	0.934 (< 0.001)
Practice (Pr)	15	0.959	3.77	0.94	3.67	−0.10	1.48	0.950 (< 0.001)
Communication (Comm)	8	0.945	3.76	1.01	3.75	−0.16	0.82	0.970 (< 0.001)
Management (Mgmt)	8	0.949	3.71	0.99	3.62	−0.13	1.00	0.963 (< 0.001)

*Note:* alpha ≥ 0.80 indicates good and alpha ≥ 0.90 excellent internal consistency [27]. Shapiro–Wilk results were significant at *N* = 205; visual inspection confirmed approximate symmetry and mild negative skew. Parametric procedures are reported alongside nonparametric robustness checks. The high composite alpha values (> 0.95 for PES‐NWI and CCNC) are acknowledged as potentially reflecting item redundancy; composite scores were used as summary measures in all primary analyses.

Figure 1 shows the distributions of NMP (NMP), practice environment scale (PES‐NWI), and CCNC scores. All three constructs demonstrated approximately normal distributions with mild negative skewness, indicating that most participants reported moderate‐to‐high levels of management practices, practice environment quality, and competency. The observed distributional patterns support the suitability of composite measures for subsequent correlational, regression, and mediation analyses.

**FIGURE 1:**
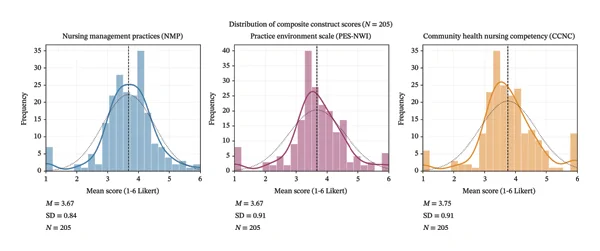
Distribution of composite construct scores for NMP, PES‐NWI, and CCNC. Dashed line = mean; solid curve = kernel density estimate; dotted curve = fitted normal density. Resolution: 300 dpi.

Table 2 presents the Pearson correlation matrix of the NMP, PES‐NWI, and CCNC subscales. All correlations were positive and statistically significant (*p* < 0.001), indicating consistent associations across management practices, practice environment dimensions, and CCNC. Among the NMP dimensions, WW demonstrated the strongest associations with CCNC subscales (*r* = 0.66–0.77), particularly with community care practice (*r* = 0.77). Within the practice environment, foundations for quality of care and nurse participation in affairs showed the strongest relationships with competency dimensions (*r* = 0.63–0.69 and *r* = 0.61–0.68, respectively). The highest correlations were observed between the CCNC subscales themselves (practice‐communication, *r* = 0.83; communication‐management, *r* = 0.83), supporting the internal coherence of the competency construct. Overall, the findings indicate strong interrelationships among NMP, the practice environment, and CCNC.

**TABLE 2:** Pearson correlation matrix of NMP, PES‐NWI, and CCNC subscales (*N* = 205).

	WLB	WD	WE	WW	Part	Found	NMgr	Staff	Coll	Pr	Comm	Mgmt
WLB	1.00											
WD	0.53	1.00										
WE	0.53	0.76	1.00									
WW	0.57	0.58	0.67	1.00								
PES‐Part	0.46	0.64	0.61	0.60	1.00							
PES‐Found	0.46	0.64	0.65	0.57	0.79	1.00						
PES‐NMgr	0.48	0.63	0.65	0.56	0.72	0.78	1.00					
PES‐Staff	0.47	0.55	0.61	0.55	0.75	0.70	0.77	1.00				
PES‐Coll	0.39	0.53	0.58	0.57	0.72	0.69	0.67	0.72	1.00			
CCNC‐Pr	0.48	0.65	0.72	0.77	0.65	0.69	0.64	0.59	0.65	1.00		
CCNC‐Comm	0.48	0.58	0.66	0.70	0.61	0.63	0.62	0.55	0.62	0.83	1.00	
CCNC‐Mgmt	0.41	0.58	0.60	0.66	0.68	0.69	0.67	0.61	0.66	0.79	0.83	1.00

*Note:* All coefficients are significant at *p* < 0.001 (two‐tailed). NMP subscales: WLB = work–life balance, WD = work design, WE = work environment, WW = work world. PES‐NWI subscales: Part = participation, Found = foundations, NMgr = nurse manager, Staff = staffing, Coll = collegial relations. CCNC subscales: Pr = practice, Comm = communication, Mgmt = management.

Table 3 presents comparisons of the NMP, PES‐NWI, and CCNC scores across gender and educational groups. Male nurses reported significantly higher scores than female nurses on both NMP (*M* = 3.88 vs. 3.59, *p* = 0.037, *d* = 0.34) and CCNC (*M* = 4.05 vs. 3.64, *p* = 0.012, *d* = 0.46), although the effect sizes were small‐to‐moderate. No significant gender difference was observed in the PES‐NWI scores (*p* = 0.073). Educational attainment was not significantly associated with NMP, PES‐NWI, or CCNC scores (all *p* > 0.05). Although nurses with MSN qualifications reported the highest mean CCNC score (*M* = 4.30), the overall group difference did not reach statistical significance (*p* = 0.069). Nonparametric analyses yielded substantively identical conclusions, supporting the robustness of the findings.

**TABLE 3:** Demographic group comparisons on composite scores (*N* = 205).

Comparison	Group	*n*	*M*	SD	Test	Statistic	*p*	Effect size
Gender‐NMP	Female	146	3.59	0.80	Welch *t* (101.7)	2.11	0.037^∗^	*d* = 0.34
	Male	59	3.88	0.90				

Gender‐PES‐NWI	Female	146	3.59	0.82	Welch *t* (89.8)	1.82	0.073 (ns)	*d* = 0.32
	Male	59	3.87	1.10				

Gender‐CCNC	Female	146	3.64	0.80	Welch *t* (89.6)	2.58	0.012^∗^	*d* = 0.46
	Male	59	4.05	1.10				

Education‐NMP	Diploma	103	3.61	0.75	*F* (2, 202)	0.92	0.402 (ns)	eta2 = 0.009
	BSN	93	3.72	0.91				
	MSN	9	3.94	1.00				

Education‐PES‐NWI	Diploma	103	3.63	0.81	*F* (2, 202)	0.56	0.572 (ns)	eta2 = 0.006
	BSN	93	3.68	0.98				
	MSN	9	3.96	1.35				

Education‐CCNC	Diploma	103	3.64	0.79	*F* (2, 202)	2.72	0.069 (ns)	eta2 = 0.026
	BSN	93	3.83	1.02				
	MSN	9	4.30	0.91				

*Note:* Cohen’s d was computed with pooled SD; eta2 for ANOVA. Nonparametric verification (Mann–Whitney U/Kruskal–Wallis H) yielded substantively identical conclusions (gender NMP U: *p* = 0.043; gender CCNC U: *p* = 0.015; education KWH all *p* > 0.15). ns = nonsignificant (*p* > 0.05).

^∗^ *p* < 0.05.

Table 4 presents the hierarchical regression models predicting CCNC. Model 1 explained 70.0% of the variance in CCNC (Adjusted *R* ^2^ = 0.685, *p* < 0.001), with WW, work environment, and work design emerging as significant positive predictors, whereas work–life balance was not significantly associated with competency. Total nursing experience was also positively associated with CCNC. In Model 2, the composite measures of NMP and the practice environment scale (PES‐NWI) were entered, jointly explaining 71.5% of the variance in CCNC (adjusted *R* ^2^ = 0.703, *p* < 0.001). Both NMP (*β* = 0.47, *p* < 0.001) and PES‐NWI (*β* = 0.38, *p* < 0.001) remained significant independent predictors. The modest increase in explained variance (Δ*R* ^2^ = 0.015) indicates that the practice environment contributed additional explanatory value beyond management practices alone. Multicollinearity diagnostics were within acceptable limits (maximum VIF = 4.2; condition indices < 30), and HC3 robust standard errors yielded conclusions consistent with conventional estimates. Although the models explained a substantial proportion of variance in CCNC, the findings should be interpreted cautiously given the self‐report and cross‐sectional design.

**TABLE 4:** Hierarchical multiple regression predicting CCNC (*N* = 202 with complete covariates).

Predictor	Model 1B (SE)	Beta	*p*	Model 2B (SE)	Beta	*p*
Constant	0.79 (0.35)	—	0.026	0.51 (0.34)	—	0.136
Work–life balance (WLB)	−0.02 (0.05)	−0.02	0.699	—		
Work design (WD)	0.16 (0.06)	0.17	0.009	—		
Work environment (WE)	0.23 (0.06)	0.25	< 0.001	—		
Work world (WW)	0.41 (0.05)	0.47	< 0.001	—		
NMP (composite)	—			0.50 (0.07)	0.47	< 0.001
PES‐NWI (composite)	—			0.38 (0.06)	0.38	< 0.001
Age (years)	−0.01 (0.01)	−0.05	0.391	−0.01 (0.01)	−0.07	0.228
Total experience (years)	0.04 (0.02)	0.15	0.020	0.04 (0.02)	0.15	0.018
Current‐role experience	−0.01 (0.02)	−0.02	0.771	0.00 (0.02)	−0.00	0.943
Education: BSN vs. diploma	0.07 (0.08)	0.04	0.353	0.14 (0.08)	0.08	0.078
Education: MSN vs. diploma	0.37 (0.18)	0.09	0.041	0.30 (0.18)	0.07	0.087
Gender: male vs. female	0.12 (0.08)	0.06	0.149	0.15 (0.08)	0.07	0.057
*R* ^2^		0.700			0.715	
Adjusted *R* ^2^		0.685			0.703	
*F* (df1, df2)		44.62 (10,191)^∗∗∗^			60.38 (8193)^∗∗∗^	
Delta *R* ^2^ (Model 2 − Model 1)					+ 0.015	
Max VIF		4.2			3.7	

*Note: B* = unstandardized coefficient; *β* = standardized coefficient. All VIF values were < 5.0, indicating no evidence of problematic multicollinearity.

Abbreviation: VIF = variance inflation factor.

^∗∗∗^ *p* < 0.001.

Table 5 presents the mediation analysis examining the role of the NPE (PES‐NWI) in the association between NMP and CCNC. NMP was positively associated with the PES‐NWI (path a: *β* = 0.760, *p* < 0.001), and the PES‐NWI remained positively associated with the CCNC after controlling for NMP (path b: *β* = 0.399, *p* < 0.001). The total association between NMP and CCNC was significant (path c: *β* = 0.789, *p* < 0.001), and although the direct association remained significant after adjustment for the PES‐NWI (path c’: *β* = 0.499, *p* < 0.001), its magnitude was reduced. Bootstrap analysis confirmed a significant indirect association (*B* = 0.331, 95% CI [0.114, 0.516]), accounting for 38.4% of the total association. These findings indicate partial statistical mediation, whereby perceptions of the practice environment account for a substantial proportion of the association between management practices and community nursing competencies.

**TABLE 5:** Mediation analysis: PES‐NWI as a statistical intermediary in the NMP‐CCNC association (*N* = 205; 5000 bootstrap resamples).

Path	Description	*B*	SE	95% CI	Beta	*p*
*A*	NMP‐ > PES‐NWI	0.831	0.050	[0.732, 0.929]	0.760	< 0.001
*B*	PES‐NWI‐ > CCNC (controlling for NMP)	0.399	0.060	[0.281, 0.518]	0.399	< 0.001
*c* (total)	NMP‐ > CCNC	0.862	0.047	[0.770, 0.954]	0.789	< 0.001
C’ (direct)	NMP‐ > CCNC (controlling for PES‐NWI)	0.531	0.066	[0.401, 0.661]	0.499	< 0.001
*a* × *b* (indirect)	NMP‐ > PES‐NWI‐ > CCNC	0.331	0.103	[0.114, 0.516] (BC bootstrap)	—	Sobel *Z* = 6.15, < 0.001
Proportion mediated	(*a* × *b*)/*c*	38.4%	—	—	—	—

*Note: B* = unstandardized coefficient; *β* = standardized coefficient; BC = bias‐corrected bootstrap confidence interval. An indirect association was considered statistically significant when the 95% bootstrap CI did not include zero.

Abbreviations: CI = confidence interval; SE = standard error.

Figure 2 illustrates the hypothesized mediation model and the bivariate relationships among NMP, NPE (PES‐NWI), and CCNC. As shown in Panel A, NMP exhibited a significant total association with CCNC, and this association was attenuated but remained significant after the inclusion of PES‐NWI, indicating partial statistical mediation. The bootstrap confidence interval for the indirect association excluded zero, supporting the significance of this mediating pathway. Panels B–D further demonstrate strong positive linear associations between NMP and CCNC (*r* = 0.789), PES‐NWI and CCNC (*r* = 0.768), and NMP and PES‐NWI (*r* = 0.760), consistent with the mediation model. Collectively, these findings suggest that more favorable perceptions of NMP are associated with higher community nursing competency, both directly and indirectly, through a more favorable practice environment.

**FIGURE 2:**
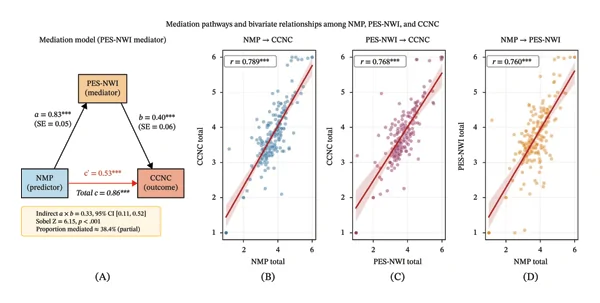
Mediation pathways and bivariate relationships among NMP, PES‐NWI, and CCNC. (A) Simple mediation model with unstandardized path coefficients; values in parentheses are standard errors. (B–D) Scatterplots with OLS regression lines and bootstrap 95% confidence bands illustrating the strong positive associations underlying the mediation model. ^∗∗∗^ *p* < 0.001. Resolution: 300 dpi.

## 6. Discussion

This study examined a theoretically derived associational model in which NMP are associated with CCNC, both directly and through the NPE, in a sample of 205 community/PHC nurses working in Saudi Arabia’s Northern Border Region. Four findings warrant this emphasis: first, the three focal constructs were strongly and positively associated (NMP<‐>NPE *r* = 0.76; NMP<‐>CCNC *r* = 0.79; NPE<‐>CCNC *r* = 0.77; all *p* < 0.001), representing large effect sizes. Second, the NMP and NPE composites together explained 71.5% of the variance in CCNC (*F* [10, 191] = 44.62, *p* < 0.001), with NMP (*β* = 0.47) and NPE (*β* = 0.38) emerging as substantial independent predictors. Third, the bootstrapped mediation analysis confirmed a statistically robust indirect pathway (*a* × *b* = 0.33; 95% CI [0.11, 0.52]; Sobel *Z* = 6.15, *p* < 0.001), with NPE accounting for approximately 38.4% of the total association between NMP and CCNC, that is, partial but substantively meaningful statistical mediation. Fourth, at the subscale level, the WW dimension of NMP (*β* = 0.47) and the foundations for quality of care dimension of the PES‐NWI exhibited the closest associations with competency, signaling that the relational and quality‐systems facets of management, rather than work–life balance per se (*β* = −0.02, ns), are most closely linked to professional capability in this setting.

Two methodological points bear emphasis before discussing substantive interpretations. The exceptionally high R2 values (0.700–0.715) are noteworthy and atypical for behavioral and organizational research. These values may in part reflect the conceptual overlap among the three constructs, the homogeneity of the single‐region sample, the high internal consistency of the instruments (potentially indicating item redundancy), and some residual common method variance. The CMV diagnostic analyses (Harman’s test first factor = 43.6%; marker‐variable delta *R* ^2^ = 0.002) suggest that CMV alone is unlikely to account for the observed effect sizes, and the VIF values (max 4.2) confirm that multicollinearity has not invalidated the regression estimates. Nevertheless, readers should interpret the *R* ^2^ values as reflecting the shared conceptual and psychometric space among these constructs in this specific sample rather than as definitive evidence of explanatory dominance. Replication with alternative instruments and independent samples is needed before strong inferential conclusions can be drawn.

### 6.1. Interpretation Against the Contemporary Evidence Base

These results are consistent with a conceptualization of community nursing competency as an organizationally contextualized professional attribute associated with both direct management inputs and the broader practice environment rather than as a purely individual characteristic. The strong direct association between NMP and CCNC, alongside the significant indirect association through the NPE, indicates that management context is associated with competency through two complementary statistical pathways: a proximal pathway (supervision, role clarity, feedback, and developmental support) and a contextual pathway (staffing adequacy, collegial relations, autonomy, and quality‐assurance climate). This dual‐pathway interpretation is congruent with and extends recent evidence demonstrating that practice environment characteristics and manager leadership style are jointly associated with collaboration and care quality in acute and primary care nurses [31] and with the Lucas et al.’s [32] finding that the NPE is positively associated with nurse‐perceived quality and patient safety in PHC.

The partial nature of statistical mediation is theoretically informative. It positions the NPE not as a passive backdrop but as an active organizational pathway through which the management context is associated with professional capability, while leaving conceptual room for management to be associated with competency through additional, nonenvironmental channels (e.g., direct mentoring, coaching, and role modeling). This is consistent with recent PHC evidence identifying people management, nurse leadership, teamwork, and quality‐assurance strategies as the most influential features of favorable practice environments [33] and with scoping‐review evidence in primary care showing that leadership, communication, and organizational culture form the connective tissue of safety‐promoting environments [34]. The present study advances this literature by shifting the dependent variable from safety culture to competency, thereby demonstrating that the explanatory reach of the NPE extends across the SPO chain. However, it is important to reiterate that the cross‐sectional design does not permit conclusions about causal direction; the model identifies associational patterns that are consistent with the Donabedian’s SPO framework but does not test causal mechanisms.

### 6.2. Contextual Significance for the Saudi PHC System

The global NPE literature originates from acute hospital‐based settings in high‐income countries; therefore, situating the management‐environment‐competency nexus within Saudi PHC in an administratively peripheral region represents a substantive contextual contribution. Recent national workforce analyses document persistent regional inequalities in the kingdom’s nursing workforce, with uneven nurse‐to‐population ratios and staffing distribution across provinces [15]. In peripheral contexts such as the Northern Border Region, where the compensatory infrastructure of metropolitan healthcare settings is absent and PHC centers serve as the de facto primary and often sole healthcare point of contact, the quality of local management and the immediate practice environment may carry disproportionate weight in shaping whether nurses can practice competently. The findings suggest that, even where macro‐workforce constraints persist, investing in unit‐level management capacity and environmental quality may represent a comparatively feasible lever for strengthening community nursing, a position directly aligned with Vision 2030 and the Health Sector Transformation Program. However, because the study is cross‐sectional and single region, these implications are directional rather than prescriptive; they should be validated through longitudinal and multisite studies before forming the basis of policy action.

Male nurses scored significantly higher than female nurses on NMP (*d* = 0.34, *p* = 0.037) and CCNC (*d* = 0.46, *p* = 0.012), with a nonsignificant trend on PES‐NWI (*d* = 0.32, *p* = 0.073). This finding warrants cautious interpretation. The nursing profession is historically and numerically female‐dominated in most global contexts, and a pattern of higher self‐reported management quality and competency among male nurses is not commonly reported in the international literature. Possible explanations specific to the Northern Border Region context include differential cultural role expectations that may influence self‐rating tendencies; organizational selection effects that concentrate male nurses in relatively better‐resourced or better‐managed units or facility types; differences in seniority distribution by gender; or culturally inflected self‐report patterns. The study design did not permit adjudication of these explanations. From a management standpoint, the productive question is not whether groups differ numerically, but which features of management and the practice environment are associated with more equitable distributions of competency expression across the workforce, a question that warrants future investigation.

Educational attainment did not yield statistically significant omnibus differences in any composite (all *p* > 0.05). The CCNC difference across education levels approached, but did not reach, statistical significance (*p* = 0.069, eta2 = 0.026). This nonsignificant trend is most parsimoniously explained by the very small MSN subsample (*n* = 9; 4.4%), which severely limits the power of the educational group comparisons. Any numerical differences in the CCNC scores across educational groups cannot be interpreted as established findings in the absence of statistical significance.

### 6.3. Strengths and Limitations

This study had several strengths. The study was guided by the Donabedian’s SPO framework, employed validated instruments with excellent internal consistency, and adhered to the STROBE reporting recommendations. The response rate was high (78.5%), and the analyses were strengthened through a priori power estimation, multicollinearity assessment, common method variance (CMV) diagnostics, and bias‐corrected bootstrap mediation procedures. In addition, the focus on community nurses working in Saudi Arabia’s Northern Border Region contributes to the evidence from an under‐researched PHC context.

Several limitations should be considered when interpreting these findings. First, the cross‐sectional design precludes conclusions regarding causality or temporal ordering, and the mediation model should be interpreted as reflecting statistical associations, rather than causal pathways. Second, all study variables were measured using self‐report questionnaires completed by the same respondents, creating the potential for common method bias, despite diagnostic analyses suggesting that CMV was unlikely to have substantially influenced the findings. Third, the very high reliability coefficients observed for some composite measures may indicate a degree of item redundancy and could have contributed to the strong intercorrelations among the study variables. Fourth, the use of convenience sampling within a single geographical region may limit the generalizability of the findings to other healthcare settings or regions in Saudi Arabia. Fifth, NMP were assessed using an adapted measure derived from the QNWL Survey, which captures management‐related aspects of the work environment but does not directly assess specific managerial behaviors or leadership styles. Finally, community nursing competency was measured through self‐assessment and may not fully correspond to objective or supervisor‐rated evaluations of competence. Future studies should employ longitudinal designs, including multiple data sources, and incorporate objective measures of competency and management performance to strengthen the evidence in this area.

## 7. Conclusion

This study identifies the NPE as a significant statistical intermediary in the association between NMP and CCNC in a Saudi PHC context. By empirically integrating management, environment, and competency within a single associational model anchored in the Donabedian’s SPO framework, it reframes quality improvement in community nursing as a relational and organizational endeavor in which the management context and practice environment are both independently and jointly associated with professional capability. These findings are consistent with theoretical propositions about the role of structural and process factors in shaping professional outcomes but do not establish causal pathways; longitudinal and multisite studies are needed to confirm directionality and test the generalizability of the associations to other PHC contexts. In the interim, the study provides a theoretically grounded and empirically supported rationale for investing in unit‐level management capacity and practice environment quality as potentially feasible strategies for advancing community nursing competency in peripheral PHC settings undergoing system‐wide reform.

### 7.1. Implications for Nursing Management

For nurse managers, directors of nursing, and PHC policymakers, the findings suggest several practical implications that should be interpreted considering the cross‐sectional design and specific context of the Northern Border Region. First, competency development may benefit from approaches that integrate workforce development with improvements in the practice environment of the latter. Such strategies could include mentorship initiatives, protected opportunities for quality improvement activities, and efforts to ensure adequate staffing and resource availability within PHC settings. Second, leadership development initiatives may help strengthen managerial capacity and foster practice environments that support professional growth and competency development. In peripheral regions where leadership pipelines may be limited, targeted professional development and leadership‐training opportunities should be considered. Third, strengthening mechanisms that support nurses’ participation in decision‐making, workforce planning, resource allocation, and quality improvement processes may contribute to more supportive practice environments for nurses. Within the context of ongoing healthcare reforms, attention to equitable access to professional development resources and the inclusion of nursing workforce perspectives in organizational planning may further support CCNC. Given the observational nature of this study, these implications should be viewed as evidence‐based recommendations that warrant further evaluation through longitudinal and implementation studies.

## Author Contributions

Fathia Ahmed Mersal, Amal Ahmed Elbilgahy, Mohamed Yehia Ali Mohamed, and Bindu Bharathi contributed to the conceptualization of the research ideas, development of research instruments, and data collection, organization, and interpretation. Fathia Ahmed Mersal, Amal Ahmed Elbilgahy, and Fadiyah Jadid Alanazi were involved in writing the introduction and drafting the Discussion section. All authors contributed to the preparation of the initial and final drafts of the manuscript and provided the logistical and administrative support.

## Funding

The authors gratefully acknowledge the approval and support of this research study by the grant No. (NBU‐FPEJ‐2026‐1041‐01) from the Deanship of Scientific Research at Northern Border University, Arar, Saudi Arabia.

## Disclosure

All authors critically reviewed, revised, and approved the final version of the manuscript and took full responsibility for its content and integrity.

## Ethics Statement

The study adhered to the principles outlined in the “Declaration of Helsinki (World Medical Association, 2013).” Ethical agreement was granted from Northern Border University with the following number HAP‐09‐A‐043 with decision number 12/26/H, 14‐02‐2026. Ethical approval was obtained from the relevant Institutional Review Board (IRB) and the Directorate of Health Affairs of the Northern Border Region before data collection. A written information sheet describing the study’s purpose, its voluntary nature, anonymity safeguards, data confidentiality, and participants’ right to withdraw without consequence preceded the questionnaire. Submission of the completed questionnaire constituted implied informed consent. No personally identifying information was collected, and electronic data were stored on password‐protected servers accessible only to the research team.

## Conflicts of Interest

The authors declare no conflicts of interest.

## Data Availability

The data that support the findings of this study are available on request from the corresponding author. The data are not publicly available due to privacy or ethical restrictions.
